# Cortical Astrocyte Progenitors and Astrocytes from Human Pluripotent Stem Cells

**DOI:** 10.3390/jpm13030538

**Published:** 2023-03-17

**Authors:** Ingrid Battistella, Alessandro Cutarelli, Jacopo Zasso, Massimo Clerici, Carlo Sala, Matteo Marcatili, Luciano Conti

**Affiliations:** 1 Laboratory of Stem Cell Biology, Department of Cellular, Computational and Integrative Biology-CIBIO, University of Trento, 38123 Trento, Italy; 2 Department of Medicine and Surgery, University of Milano Bicocca, 20900 Monza, Italy; 3 Department of Mental Health, Fondazione IRCCS San Gerardo dei Tintori, 20900 Monza, Italy; 4 National Research Council Neuroscience Institute, 20100 Milan, Italy

**Keywords:** induced pluripotent stem cells, human astrocyte cortical progenitors, human cortical astrocytes

## Abstract

Astrocytes coordinate several homeostatic processes of the central nervous system and play essential roles for normal brain development and response to disease conditions. Protocols for the conversion of human induced pluripotent stem cells (hiPSCs) into mature astrocytes have opened to the generation of *in vitro* systems to explore astrocytes’ functions in living human cell contexts and patient-specific settings. In this study, we present an optimized monolayer procedure to commit hiPSC-derived cortical progenitors into enriched populations of cortical astrocyte progenitor cells (CX APCs) that can be further amplified and efficiently differentiated into mature astrocytes. Our optimized system provides a valid tool to explore the role of these cells in neurodevelopmental and neuropsychiatric diseases, opening it up to applications in drug development and biomarkers discovery/validation.

## 1. Introduction

Glial cells represent the most abundant cell type within the central nervous system (CNS), forming, depending on the region considered, between 33 and 66% of the total brain mass [1]. They embody a heterogeneous population classified into different subgroups based on morphology and function. These subgroups comprise macroglia that includes astrocytes, oligodendrocytes and Schwann cells, and microglia that includes cells originating from the immune system. Among the overall glial populations, astrocytes represent the most abundant component, and constitute up to 20–50% of the brain volume [2,3]. Unlike neurons, astrocytes are not electrically excitable. Nevertheless, they play multiple roles in the CNS physiology. Within the human brain, astrocytes have different essential roles including support and guidance for migrating neurons, regulation of the cerebral blood flow [4] and maintenance of neurotransmission homeostasis [5]. In addition, astrocytes are involved in the inflammatory response, in blood brain barrier formation and in synapse regulation [6] and plasticity [7].

Noteworthy, astroglial abundance, complexity and pleomorphism have increased significantly with mammalian evolution, which suggests an association between human glial evolution and the development of human-selective neuropsychiatric disorders, including schizophrenia (SCZ), major depression disorder (MDD) and bipolar disorders (BP) [8,9,10].

Recent studies performed on human *post-mortem* brain tissue have shown that alterations in the astrocyte number and functionality are associated to both SCZ and MDD conditions. In particular, astrocyte density, number, and morphology have been shown to vary significantly in SCZ patients depending on the brain region studied [11,12]. Instead, the inspection of *post-mortem* brains from MDD patients revealed a reduction in glial cell count and cell density [13,14]. However, advancements in dissecting astroglial roles and their contribution in the development and progression of mental disorders have been largely limited by the difficulty to access live/functional human brain tissue. Notably, transcriptome-wide profiling studies have revealed differences between murine and human astrocytes, thus implying limitations to exploit mouse models in this context [15].

Human induced pluripotent stem cell (hiPSC) technology has overcome these limitations, opening new avenues to study glia development and dysfunction in neuropsychiatric disorders based on live patient-specific cell populations.

In recent years, several methods have been developed for astrocytes production from hiPSCs to generate *in vitro* cell models of neuropsychiatric disorders [16,17,18,19] or for in vivo transplantation studies [8,16,20,21].

Nevertheless, most of the existing methods are time-consuming (requiring up to 5–6 months) [17,22] and exhibit a high variability in terms of efficiency and/or require sorting to reduce variability [23]. In addition, protocols reported are commonly based on embryoid bodies/floating steps and on the use of serum-supplemented media [24,25,26]. Collectively, these considerations highlight the need for the field to develop rapid and efficient monolayer and serum-free protocols for the generation of hiPSC-derived astrocytes.

Here, we present an *in vitro* optimized monolayer procedure to generate enriched populations of cortical astrocyte progenitor cells (CX APCs) that can be amplified and further efficiently differentiated into mature astrocytes by using xeno-free chemically-defined conditions.

Altogether, our optimized differentiation protocol enables the characterization of differentiating hiPSC-derived cortical APCs and mature astrocytes, and will serve as a robust platform to exploit for drug development and biomarkers discovery/validation approaches in neurodegenerative and neuropsychiatric disorders.

## 2. Materials and Methods

### 2.1. Cell Culture

hiPSCs used in this study were purchased from ThermoFisher Scientific (Monza, Italy) and were verified for their pluripotency and normal karyotype. The cells were maintained in feeder-free conditions in Essential 8 (E8) medium as previously reported [27,28,29]. For passaging, 70–80% confluent cultures were rinsed twice with DPBS without Ca^2+^/Mg^2+^, detached with dissociation solution and then dissociated, collected and centrifuged at 200× *g* for 5 min. The resulting pellet was resuspended in E8 medium and 10^4^ cells/cm^2^ were plated as previously reported [27,28,29]. Cells were cultured in 5% CO_2_ at 37 °C. All cell culture media and reagents were obtained from ThermoFisher Scientific.

### 2.2. Cortical Neural Progenitors, Cortical Astrocyte Progenitors and Astrocyte Differentiation

hiPSC-derived cortical progenitors were differentiated as previously described [30]. Briefly, hiPSCs were committed to a neural lineage and subsequently to the dorsal telencephalic lineage by using N2B27 medium supplemented with 4 ng/mL human recombinant Fibroblast Growth Factor-2 (Peprotech, London, UK), 500 ng/mL Noggin (Peprotech, London, UK) and 20 mM SB431542 (Santa Cruz Biotechnologies, Heidelberg, Germany). Cells were then seeded on poly-D-Lysine and laminin-coated plastic dishes (Sigma-Aldrich, Milan, Italy) in medium supplemented with 10 μM Rock inhibitor Y27632 (Santa Cruz Biotechnologies, Heidelberg, Germany). At day 10, neural rosettes containing cortical progenitor cells were collected and plated on poly-D-Lysine/laminin-treated culture dishes in N2B27 medium containing 10 ng/mL Epidermal Growth Factor (EGF; Peprotech, London, UK), 10 ng/mL Fibroblast Growth Factor-2 (FGF-2; Peprotech, London, UK) and 20 ng/mL Brain-Derived Neurotrophic Factor (BDNF; Peprotech, London, UK). Confluent cultures were passed as small multicellular clumps at a ratio of 1:3 using StemPro Accutase (Sigma-Aldrich, Milan, Italy) and amplified until passages 8–10.

**Figure 1:**
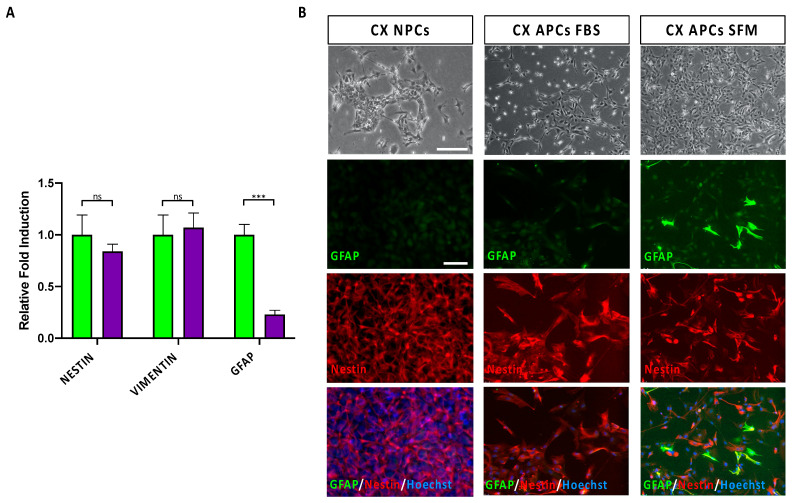
CX hiPSC-derived NPCs were committed to APC identity after DIV 25 of exposure to ACM-SFM or ACM-FBS. (**A**) qPCR analysis for NESTIN, VIMENTIN and GFAP transcript levels in cultures of CX NPCs exposed for DIV 25 to ACM-SFM (green bars) or ACM-FBS (purple bars). Values were normalized to GAPDH and represent the fold-change ratio related to ACM-SFM samples. Data are expressed as means ± STDV (*n* = 3 biologically independent experiments). *p* < 0.001 (***) and not significant (ns) according to *Student’s t-test* analysis. (**B**) Representative phase contrast and immunofluorescence pictures of the starting (DIV 0) CX NPCs and the resulting CX APCs at DIV 25 of exposure to ACM-SFM or ACM-FBS conditions. The fluorescence images are related to the GFAP and Nestin markers and the superimposition of these are shown. Nuclei are stained with Hoechst. Scale bar: 100 μm.

CX NPCs were exposed for 25 days to specific culture conditions to induce their gradual commitment into CX Astrocyte Progenitor Cells (CX APCs). During this time, cells kept proliferating and cultures were propagated by dissociation to single cells with StemPro Accutase (Sigma-Aldrich, Milan, Italy) every 3 days at 1:2 or 1:3 ratio based on cell density. Cultures were plated on Vitronectin-treated plastic (ThermoFisher Scientific). In this process, two different astrocyte committing media (ACM) were tested (Figure 1).

(i) ACM containing fetal bovine serum (ACM-FBS) composed of Neurobasal (ThermoFisher Scientific) and DMEM/F12 (ThermoFisher Scientific) at 1:1 ratio, supplemented with 0.5% N2 (ThermoFisher Scientific), 1% B27 (ThermoFisher Scientific), 20 ng/mL FGF-2, 20 ng/mL EGF and 1% FBS (ThermoFisher Scientific);

(ii) Serum-free based ACM (ACM-SFM) composed of Neurobasal, DMEM/F12 at 1:1 ratio, 0.5% N2, 1% B27, 20 ng/mL FGF-2, 20 ng/mL EGF.

To induce gradual maturation into astrocytes, CX APCs were dissociated to single cells with StemPro Accutase and seeded (2 × 10^4^ cells/cm^2^) on Vitronectin-coated plastic in astrocyte maturation media (AMM). In addition, in this maturation process, two diverse media were tested (Figure 1):

(i) AMM containing fetal bovine serum (AMM-FBS) composed of DMEM/F12 (at 1:1 ratio, supplemented with 1% N2 and 5% FBS;

(ii) AMM Serum-free medium (AMM-SFM) composed of DMEM/F12 supplemented with 1% N2, 10 ng/mL CNTF (Peprotech, London, UK) and 10 ng/mL BMP4 (Peprotech, London, UK).

The medium was completely renewed twice a week. When cells reached confluency, cultures were passaged 1:2 by means of Accutase dissociation. CX APCs exposed to these conditions can be long-term cultured (up to 40 days were tested in this study) to gradually induce full maturation into CX Astrocytes.

### 2.3. Immunofluorescence Assay

Cultures were washed with PBS, fixed at room temperature (RT) with 4% PFA for 15 min, then exposed to permeabilizing solution (0.5% Triton X-100 in PBS) for 15 min at RT and then processed for immunofluorescence assay as previously reported [27]. Briefly, samples were then exposed to blocking solution (5% FBS, 0.3% Triton X-100 in PBS) for 1 h at RT. Cells were incubated overnight at 4 °C with the specific primary antibodies prepared in antibody solution (2% FBS, 0.2% Triton X-100 in PBS). The following day, samples were washed with PBS and then exposed for 2 h at RT to specific secondary antibodies. In this study, the following primary antibodies were used: polyclonal anti-GFAP (1:500; DAKO), monoclonal anti-Nestin (1:500; RD System), polyclonal anti-SOX2 (1:300; Millipore), polyclonal anti-Phospho Histone H3 (1:300; Cell Signaling) and monoclonal anti-TuJ1 (1:1000; Santa Cruz Biotechnology). The secondary antibodies were Alexa Fluor 568 and Alexa Fluor 488 (both 1:500; ThermoFisher Scientific). To reveal the nuclei, cultures were stained with Hoechst 33342 (ThermoFisher Scientific). Negative controls were produced by omitting the primary antibodies. Samples were inspected on a Leica DM IL LED microscope and images taken by means of a Leica DFC450 C camera. To evaluate the amount of immunoreactive cells, we considered 5–8 picture fields for each sample (at least 2 × 10^3^ cells per sample; samples were in triplicate) and analyzed them by manual counting or by using Image J software.

### 2.4. Gene Expression Analysis

Total RNA was obtained by using TriFast (Euroclone) and afterwards used for cDNA synthesys by iScript kit (Bio-Rad). For the PCR reaction, EuroTaq (EuroClone) and the specific primers were used for the amplification of the GFAP and housekeeping GAPDH transcripts. The reaction steps were 3 min at 95 °C, 30 s at 94 °C for the denaturation of the cDNA, 30 s at 60 °C for annealing, 72 °C for elongation with Taq polymerase and, finally, 72 °C for 5 min. Thirty-five cycles were performed. The PCR products (amplicons) were separated on 2% agarose gel in TAE. To allow visualization of the products to the transilluminator, ClearSight (Atlas) was added to the agarose gel. GeneRuler 1 kb Plus (ThermoFisher) was used as a molecular marker.

The reaction mix for qPCR assay included primers (200 nM), SsoAdvanced SYBR green (Bio-Rad), cDNA (2.5 ng/μL) and nuclease-free water. The RealTime System CFX96TM (Bio-Rad) was used to perform the amplification reaction. Amplification program: 95 °C for 15 min, 40 cycles (15 s 91 °C, 30 s 60 °C and 31 s 65 °C) followed by 60 cycles (5 s/cycle) from 65 °C to 95 °C with increasing the temperature (0.5 °C per cycle). GAPDH was used as a housekeeping gene.

The list and the sequences of primer used in this study are detailed in Table 1.

### 2.5. Statistical Analyses

Data are presented as mean ± STDV of duplicate samples. These are representative of at least 3 experiments performed independently. The statistic was accomplished with a dedicated GraphPad Prism package. Various groups were statistically compared with a simple variance analysis (ANOVA) and corrected for multiple LSD tests. The comparative statistical analysis for independent samples was performed with a *Student’s t-test* using Bonferroni *post hoc* test or ANOVA and *post hoc* Tukey test. The values were considered statistically significant for *p* < 0.05 (*), *p* < 0.01 (**), *p* < 0.001 (***) and *p* < 0.0001 (****).

## 3. Results

### 3.1. Specification of Cortical Astrocyte Progenitor Cells from hiPSCs

The production of human cortical astrocytes from hiPSCs represents a valuable path for exploring the contribution of this cell type to neurodegenerative and neuropsychiatric disorders and to uncover potential novel biomarkers.

In order to obtain astrocyte progenitors and mature astrocytes, cortical neural progenitors (CX NPCs) need to be exposed to conditions that impose the commitment to astrocyte lineage. In this study, the astrocyte commitment was achieved by means of a two-step process consisting of a first phase of expansion and specification, which converted CX NPCs into astrocyte precursors (CX APCs), and a second phase of astrocyte differentiation/maturation, which allowed CX APCs to differentiate into populations enriched in mature CX astrocytes (Supplementary Figure S1).

To optimize the above-mentioned processes, the effects of different culture conditions were tested. Initially, to establish a robust and simplified monolayer procedure for efficient commitment of cortical regionalized CX NPCs into CX APCs, hiPSC-derived CX NPCs were exposed for a 25-day period either to a xeno-free serum-free environment (SFM) or to fetal bovine serum-supplemented media (FBS), both supplemented with EGF and FGF-2 (for the composition of the media, see Materials and Methods section).

Both conditions induced progressive morphological changes in the cultures, leading to the appearance of cells with larger size and more fibroblast-like morphology when compared to the original bipolar NPCs. In addition, analysis of the dividing cells in culture, by the examination of pHH3 immunoreactive cells, showed that FBS-supplemented growth conditions resulted in decreased proliferative capacity, while SFM conditions ensured an efficient degree of cell proliferation (Supplementary Figure S2).

To compare the relative efficiency of these different culture conditions in driving astrocyte commitment in CX NPCs, we assessed by qPCR analysis the expression levels of transcripts for human APC markers Nestin, Vimentin and GFAP (Figure 1A). These markers belong to the intermediate filament proteins and are dynamically regulated during gliogenesis [31,32]. The analysis highlighted no significant differences in levels of Nestin and Vimentin transcripts but showed a three-fold increase in GFAP expression levels in ACM-SFM culture conditions, possibly indicating a more pronounced acquisition of APC identity. To obtain qualitative data at a single cells level, we performed an immunofluorescence analysis for GFAP, a cytoskeletal protein expressed by APCs and astrocytes, with higher expression levels in astrocytes. Together with GFAP, we evaluated the co-expression of Nestin, an intermediate filament protein of the cytoskeleton highly expressed in NPCs and APCs. Based on the expression of these two markers, we evaluated the level of commitment and differentiation of the CX NPCs into APCs and astrocytes. In fact, a strong immunoreactivity for GFAP in the absence of the Nestin immunoreactivity (i.e., GFAP^+^/Nestin^−^ cells) were indicative for the identity of a mature astrocyte [33,34]. Conversely, the co-expression of the two markers (with moderate GFAP levels; i.e., GFAP^+^/Nestin^+^ cells) could indicate that the cell is an element of radial glia and potentially an astrocyte precursor [33,34]. Finally, the presence of Nestin immunoreactive signal in the absence of GFAP (i.e., GFAP^−^/Nestin^+^ cells) identified a cell as an NPC not committed to astrocyte lineage [33,34]. The immunofluorescence analysis showed the presence of immunoreactive cells for these markers, although with different levels of signal and percentage of positive cells (Figure 1B).

Specifically, cells in both conditions homogeneously exhibited Nestin immunoreactivity. Notably, CX APCs maintained in ACM-SFM or ACM-FBS highlighted a reduced presence of GFAP^+^/Nestin^−^ cells (3.1% and 0.1%, respectively), thus indicating that only a minor fraction of the cells had undergone astrocyte maturation (Figure 1B). Most of the cells in culture co-expressed Nestin and GFAP (the latter albeit at low levels, especially in the CX APCs in FBS conditions), indicating that these two populations potentially achieved a good astrocyte commitment. However, since CX APCs cultured in ACM-FBS showed little propensity for expansion (and therefore reduced amount of material), we excluded this condition from subsequent analyses.

To note, we found that CX APCs, once specified, could be further expanded in the same SFM conditions for at least five passages without loss of proliferative capacity (not shown). These expansion steps also permit obtaining large batches of APCs that could be directly exploited for experimental needs or frozen and thawed with over 90–95% recovery of viable cells.

### 3.2. Astrocyte Commitment and Maturation of hiPSC-Derived CX APCs

**Figure 2:**
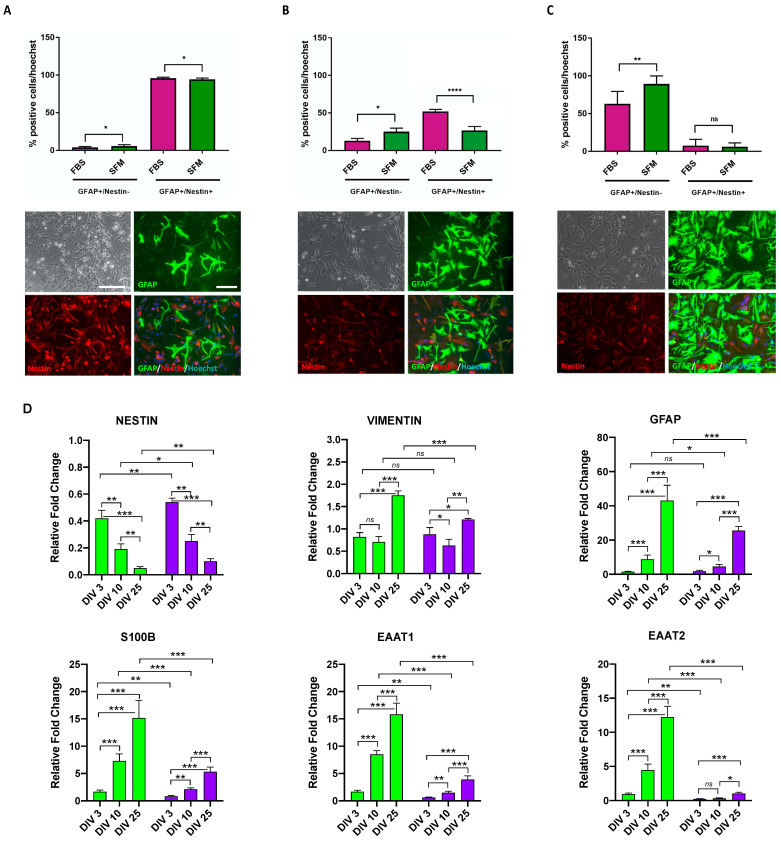
CX APCs derived in ACM-SFM conditions gradually differentiated into CX Astrocytes at DIV 25 of exposure to AMM-SFM or AMM-FBS. (**A**–**C**) Upper panel: graph showing the percentage of GFAP^+^/Nestin^−^ (astrocytes) and GFAP^+^/Nestin^+^ (APCs) cells at DIV 3 (**A**), 10 (**B**) and 25 (**C**) of exposure to AMM-FBS (purple bars) or AMM-SFM (green bars). The percentage was normalized over the total number of cells as assessed by Hoechst staining. Data are expressed as the means ± STDV (*n* = 3 biologically independent experiments). *p* < 0.05 (*), *p* < 0.01 (**), *p* < 0.0001 (****) and not significant (*ns*) according to *Student’s t-test* analysis. Lower panels: Representative phase contrast phase and immunofluorescence pictures of the CX APC cultures exposed for 3 (**A**), 10 (**B**) and 25 (**C**) DIV to AMM-SFM. The fluorescence images are related to the GFAP and Nestin markers and the superimpositions of these are shown. Nuclei are stained with Hoechst. Scale bar: 100 μm. (**D**) qPCR analysis for NESTIN, VIMENTIN, GFAP, S100B, EAAT1 and EAAT2 transcript levels in hiPSC-derived CX APCs in proliferating SFM conditions and CX APCs exposed for 3, 10 and 25 DIV to astrocyte maturation in SFM (green bars) or FBS (purple bars) conditions. Values were normalized to GAPDH and represent the fold change ratio value related to CX APCs in ACM-SFM (DIV 0). Data are expressed as the means ± STDV (*n* = 3 biologically independent experiments). *p* < 0.05 (*), *p* < 0.01 (**), *p* < 0.001 (***) and not significant (*ns*) according to ANOVA and *post hoc* Tukey test.

After having selected the ACM-SFM condition as optimal for astrocyte commitment, we exposed CX APCs to defined culture conditions to allow astrocyte maturation. In this regard, two different media were compared by exploiting either the use of an FBS-supplemented medium (AMM-FBS) or a SFM supplemented with CNTF and BMP4 (AMM-SFM; Supplementary Figure S1). SFM CX APCs were exposed to the above-mentioned conditions, and the progressive appearance of mature astrocytes in culture was assessed at DIV 3, 10 and 25 by immunofluorescence assay for the expression of GFAP and Nestin (Figure 2A and Supplementary Figure S3). For the quantification, we focused on the analyses of the GFAP^+^/Nestin^−^ (representing mature astrocytes) and GFAP^+^/Nestin^+^ (indicating the still immature APCs) cell categories. GFAP^−^/Nestin^+^ (non-astrocyte progenitors) cells were not considered as these were extremely few (less than 1%).

The percentage of GFAP^+^/Nestin^−^ cells gradually increased with time in all conditions considered, while the number of GFAP^+^/Nestin^+^ cells in cultures declined, thus indicating the progressive astrocyte maturation of the cells (Figure 2A–C and Supplementary Figure S3). Notably, the SFM condition proved to be more efficient with respect to FBS-supplemented medium in producing mature astrocytes (percentage of GFAP^+^/Nestin^−^ cells; 88 ± 9 and 63 ± 14, respectively). qPCR assay for APC and astrocyte transcripts performed on samples at different stages along the whole process of differentiation showed that NESTIN transcript levels decreased over time, with all of the astrocyte transcripts’ levels exhibiting an opposite pattern of expression (Figure 2D). This analysis further confirmed the superior astrocyte maturation effects of the AMM-SFM medium with respect to AMM-FBS conditions.

### 3.3. Time-Dependent Increased Maturation of hiPSC-Derived CX Astrocytes

**Figure 3:**
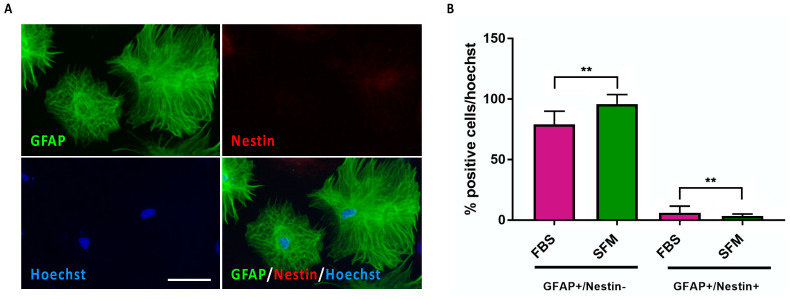

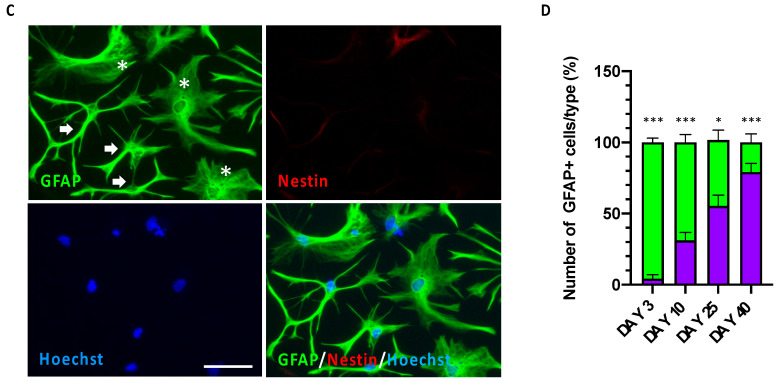
Long-term maintained in culture CX Astrocytes exhibited time-dependent maturation. (**A**) Representative immunofluorescence pictures of CX APC cultures at DIV 40 of exposure to AMM-SFM. Cultures were stained for Nestin and GFAP expression. Nuclei were stained with Hoechst. Scale bar: 75 μm. (**B**) Graph showing the percentage of GFAP^+^/Nestin^−^ (astrocytes) and GFAP^+^/Nestin^+^ (APCs) cells in DIV 40 cultures exposed to AMM-FBS (purple bars) or AMM-SFM (green bars). The percentage was normalized over the total number of cells assessed by Hoechst staining. Data were expressed as the means ± STDV (*n* = 3 biologically independent experiments). *p* < 0.01 (**), and not significant (ns) according to *Student’s t-test* analysis. (**C**) Representative immunofluorescence pictures of CX APC cultures at DIV 40 of exposure to AMM-SFM in which it is possible to observe the two typical morphologically distinct flat fibrous-like morphology and flat fibrous-like astrocyte populations. Cultures were stained for Nestin and GFAP expression. Nuclei were stained with Hoechst. Asterisks indicated astrocytes with flat fibrous-like morphology; arrows indicated astrocytes with protoplastic/reactive morphology. Scale bar: 75 μm. (**D**) Graphs reporting the number of GFAP^+^/Nestin^−^ (astrocytes) cells with flat fibrous-like morphology (purple) or with protoplastic/reactive morphology (green) in CX APC cultures exposed for 3, 10, 25 and 40 DIV to AMM-SFM. The percentage was normalized over the total number of GFAP^+^/Nestin^−^ (astrocytes) cells. Data were expressed as the means ± STDV (*n* = 3 biologically independent experiments). *p* < 0.05 (*), *p* < 0.001 (***) and not significant (ns) according to *Student’s t-test* analysis.

Cultures at DIV 25 of differentiation in SFM conditions presented an efficient generation of mature astrocytes. Next, we tested if longer maintenance in differentiative conditions could further increase the maturation of the astrocytes in culture (i.e., increased fraction of GFAP^+^/Nestin^−^ cells). At DIV 40 of exposure to AMM-SFM conditions, most of the cells in culture were strongly immunoreactive to GFAP and negative for Nestin (Figure 3A). Quantitative analysis showed that at DIV 40, the fraction of mature astrocytes, exhibited a further increase with respect to DIV 25 cultures (percentage of GFAP^+^/Nestin^−^ cells; 95 ± 7 and 88 ± 9, respectively; Figure 3B). In addition, at this stage, we found a further reduction of CX APCs in culture (percentage of GFAP^+^/Nestin^+^ cells; DIV 25: 5.4 ±4.7, 40 DIV: 1.8 ± 0.9; Figure 3B), indicating that the optimized SFM conditions efficiently favored astrocyte commitment and strongly promoted astrocyte maturation. Notably, extending the time of permanence in culture elicited a further maturation also in cultures maintained in AMM-FBS conditions (percentage of GFAP^+^/Nestin^−^ cells; DIV 40: 81 ± 11, DIV 25: 63 ± 14; Figure 3B).

The loss of APC identity and the acquisition of mature astrocyte fate of DIV 40 cultures maintained in SFM differentiative conditions was confirmed by qPCR assay showing that the transcript levels of Nestin were further decreased and those of all astrocyte markers further increased (Supplementary Figure S4). Again, the results confirmed a higher performance of astrocyte maturation promoted by SFM medium with respect to FBS conditions (Supplementary Figure S4).

It can also be noted that with progressive *in vitro* maturation, also the morphology of the cells in culture varied, with typical protoplasmic and fibrous morphologies typically detectable in different parts of the brain. Indeed, while CX APCs exhibited a characteristic radial shape (Figure 1B and Figure 2A), during the first period of differentiation/maturation the majority of GFAP^+^/Nestin^+^ cells displayed an elongated protoplastic/reactive morphology (Figure 2B,C and Figure 3C,D). To note, in cultures at DIV 40 of exposure to AMM-SFM conditions, most of the astrocytes displayed a typically flat fibrous-like astrocyte morphology with few astrocytes exhibiting an elongated protoplastic/reactive morphology (Figure 3C).

## 4. Discussion

In the recent years, the possibility to derive astrocytes from patient-specific hiPSC lines has generated increasing interest for investigating astrocytes’ contributions in the etiology and pathophysiology of neuropsychiatric diseases, including their potential involvement in the clinical response to drugs [8,9,10,35].

Here, we have reported a procedure that allows the efficient conversion of hiPSC-derived cortical NPCs in enriched cultures of human CX APCs with 25 days in monolayer fully-defined conditions and with a minimal use of small molecules. These CX APCs can be further expanded to produce large batches of CX APCs, maintaining their original capability to mature in culture into astrocytes.

In the reported conditions, the astrocyte cultures can be maintained for at least for 2 months (not shown), thus providing a useful system to investigate the molecular processes controlling progressive functional maturation. This system also allows to inspect the potential astrocyte-specific alterations that may underly the onset/progression of brain pathologies and the functional interactions of astrocytes with neurons.

To our knowledge, our method is different from most commonly used approaches that rely on 3D cultures such as embryoid bodies and neurospheres and thus require more complex and time-consuming procedures [16,17,18,19,20,21,22,33,34]. In this respect, although adherent cultures might be considered less relevant to the natural developmental process characterized by abundant intercellular interactions occurring in the complex tissue, owing to the simplicity of the protocol, it allows ease in simultaneous differentiation from several patient-specific hiPSC lines. Furthermore, since CX APCs can be extensively expanded and cryopreserved, this approach might be applicable for large studies including several patients, such as for cohorts of patients with idiopathic neuropsychiatric disorders [19,25,26,33,36,37,38,39]. Notably, although some studies have reported that the expression of GFAP may be lower in astrocytes generated from adherent cultures compared to 3D systems [32,33,39], we found here that a large percentage of astrocytes in our cultures expressed GFAP already at DIV 25. Although other protocols have reported the generation of similar enriched astrocyte populations in terms of GFAP transcript levels and percentage of GFAP-positive cells, compared to most of these studies, our protocol does not require prolonged culture periods (in some cases more than 180 days) and/or serial sorting procedures [17,22,37,40].

The reduced complexity of the differentiation strategy coupled to the use of fully-defined media with the use of only two distinct serum-free media formulations (ACM-SFM and AMM-SFM) and a limited number of factors needed for gliogenic commitment/astrocyte maturation provide a fast and accessible method to be exploited for human CNS disease modelling studies [26,39,41].

Given these advantages, the present procedure potentially offers a valid alternative that may be tailored for GMP conditions for translational approaches and for studies that require large amounts of nearly well-defined target cell populations, including biochemical analyses and HTS systems [26,39,41].

Although our protocol has the value of generating nearly homogeneous populations of human astrocytes, this homogeneity might elicit unwanted effects on the astrocyte functionality. Indeed, previous studies have shown the critical function played by neurons on astrocyte differentiation by modulating astrocytes’ morphology and the expression/localization of glutamate transporters in human astrocytes [42,43,44]. In this respect, the astrocytes in our culture conditions show diverse morphologies, which recall both the protoplasmic and fibrous astroglia types present in the human brain [45]. In the human brain, as well as in the primates and rodents, protoplasmic astrocytes are the most abundant astrocyte type. Protoplasmic astrocytes have been shown to interact with an exceptional number of synapses in the human brain, and thus are considered as important players in modulating inter-neuronal communication and for local integration of information from a large number of synapses. On the other hand, fibrous astroglia differs from protoplasmic astrocytes as they have a larger size, are less branched and straightened, and perform fewer fine processes, and can be mainly found in the cerebral cortex white matter [46]. Fibrous astrocytes have been proposed, due to the lack of synapses in the white matter, to play a general role in metabolic support but not in the modulation of neuronal activity. In our improved conditions, the fraction of astrocytes with fibrous-like morphology in our cultures progressively increases along with maturation time, with nearly 80% of GFAP^+^ astrocytes displaying a flat fibrous-like astrocyte morphology at DIV 40 (Figure 3A,C,D). This bias might be linked to the absence of contaminating neurons in our mature astrocyte cultures, thus favoring the acquisition of a fibrous-like rather than protoplastic/reactive morphology/identity. To note, some groups have reported a similar fibrous-like morphology of the mature astrocyte cultures TCW. Nevertheless, other groups have reported the appearance of mature GFAP-positive astrocyte cultures with the same type of morphologies we have seen here but with different proportions [17,33,35,47]. In this regard, we can speculate that these discrepancies might be due to different media compositions used for astrocyte maturation that incorporate growth factors/cytokines that might favor a more reactive astrocyte phenotype rather than a quiescent status [33].

Future efforts should be devoted to investigation of the maintenance of the cortical identity in mature astrocyte cultures and to co-culturing neurons with astrocytes (and other cell types) to closely reproduce a completely controlled human-relevant environment able to address issues of reciprocal maturation and to study glial cells implication/contribution in healthy and pathological conditions.

## 5. Conclusions

In the present study, we report a culture procedure to generate CX APCs and mature astrocytes from hiPSCs. Additional studies are required to demonstrate the functional characteristics of these astrocytes, including glutamate uptake, spontaneous calcium oscillation and their effects on neuronal maturation. The availability of CX astrocytes obtained by means of the present procedure may help to address the cell-autonomous and non-cell-autonomous contribution of astrocytes to the onset/progression of CNS diseases. These cells can also represent a new tool for neuropsychiatric diseases modeling and biomarkers discovery and to test candidate drugs in an *in vitro* human-specific context.

## Figures and Tables

**Table 1:** Sequences of the primers employed in this study.

Gene	Fw Primer	Rev Primer
hNESTIN	GGAGAAGGACCAAGAACTG	ACCTCCTCTGTGGCATTC
hGFAP	CTGGAGAGGAAGATTGAGTCGC	ACGTCAAGCTCCACATGGACCT
hALDOLASE C	CATTCTGGCTGCGGATGAGTCT	CACACGGTCATCAGCACTGAAC
hEAAT1	GGTTGCTGCAAGCACTCATCAC	CACGCCATTGTTCTCTTCCAGG
hEAAT2	TGCCAACAGAGGACATCAGCCT	CAGCTCAGACTTGGAGAGGTGA
hVIMENTIN	TGGACCAGCTAACCAACGAC	GCCAGAGACGCATTGTCAAC
hS100B	TGTAGACCCTAACCCGGAGG	TGCATGGATGAGGAACGCAT
hGAPDH	CCACTCCTCCACCTTTGAC	ACCCTGTTGCTGTAGCCA

## Data Availability

The data presented in this study are available in the article.

## Supplementary Materials

The following supporting information can be downloaded at https://www.mdpi.com/article/10.3390/jpm13030538/s1; Supplementary Figure S1: hiPSCs induction into CX NPCs, CX APCs and CX Astrocytes in monolayer conditions; Supplementary Figure S2: Proliferation potential of APCs expanded in FBS medium is significantly affected compared to SFM conditions; Supplementary Figure S3: CX APCs derived in ACM-SFM conditions gradually differentiated into CX Astrocytes upon exposure to AMM-FBS; Supplementary Figure S4: CX APCs undergo astrocyte differentiation/maturation at DIV 40 of exposure to SFM and FBS culture conditions.
