# Supplementary material for: Cortical Astrocyte Progenitors and Astrocytes from Human Pluripotent Stem Cells
Source: J Pers Med. 2023 Mar 17;13(3):538. doi: 10.3390/jpm13030538 (PMC10051695; doi:10.3390/jpm13030538)
Supplement: Supplementary file 1 [file jpm-13-00538-s001.zip › jpm-2253951-supplementary.pdf]

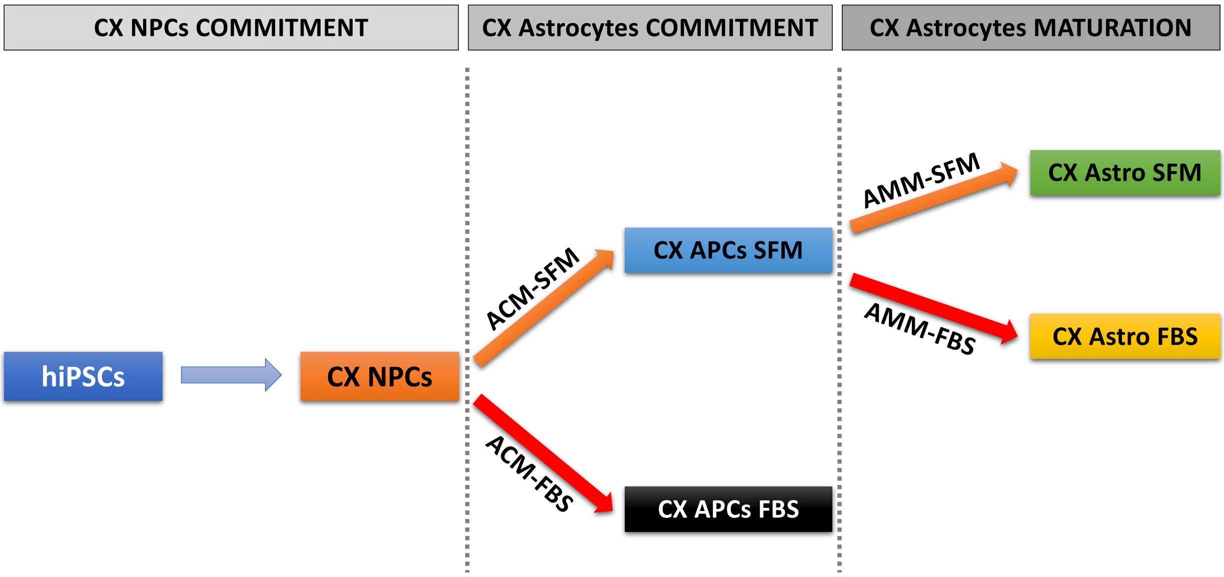


**Supplementary Figure S1.** hiPSCs induction into CX NPCs, CX APCs and CX Astrocytes in monolayer conditions. Schematic representation of the entire differentiation process and conditions tested.

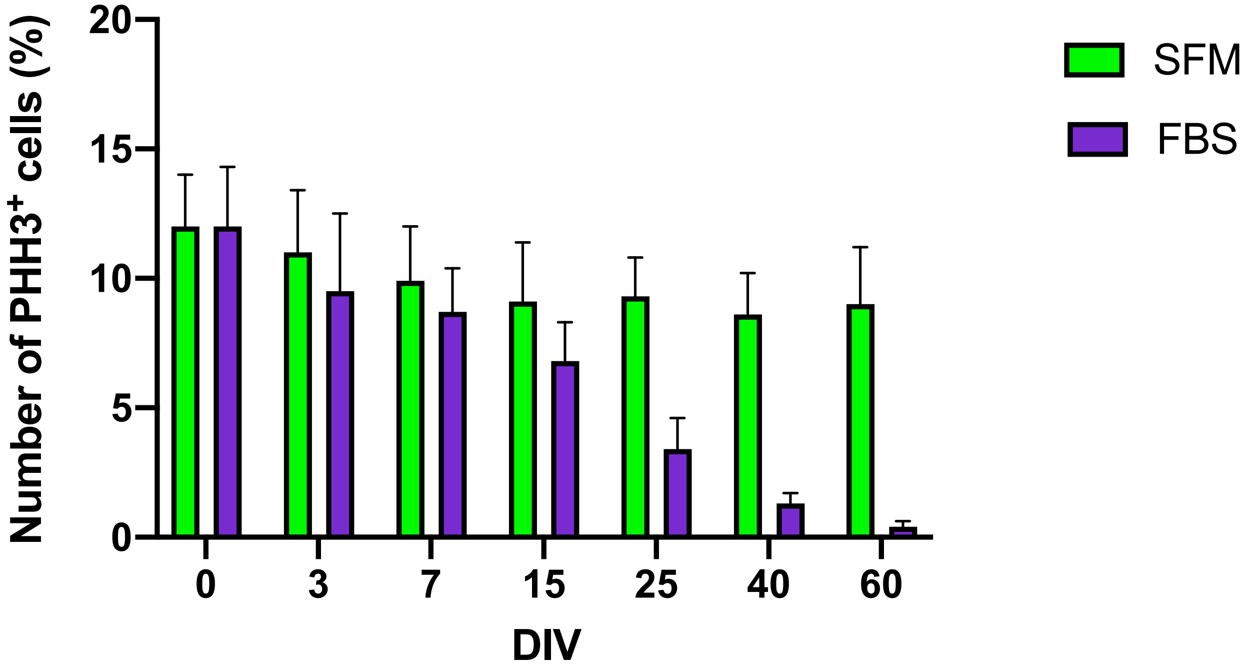


**Supplementary Figure S2.** Proliferation potential of APCs expanded in ACM-FBS medium is significantly affected compared to ACM-SFM conditions. Graph showing the number of dividing cells (phase M) at 0, 3, 7, 15, 25, 40, 60 DIV of exposure to ACM-SFM or ACM-FBS assessed by immunoreactivity for phospho Histone H3 (PHH3) marker over the total number of cells. Data are expressed as the means ± STDV (n = 3 biologically independent experiments).

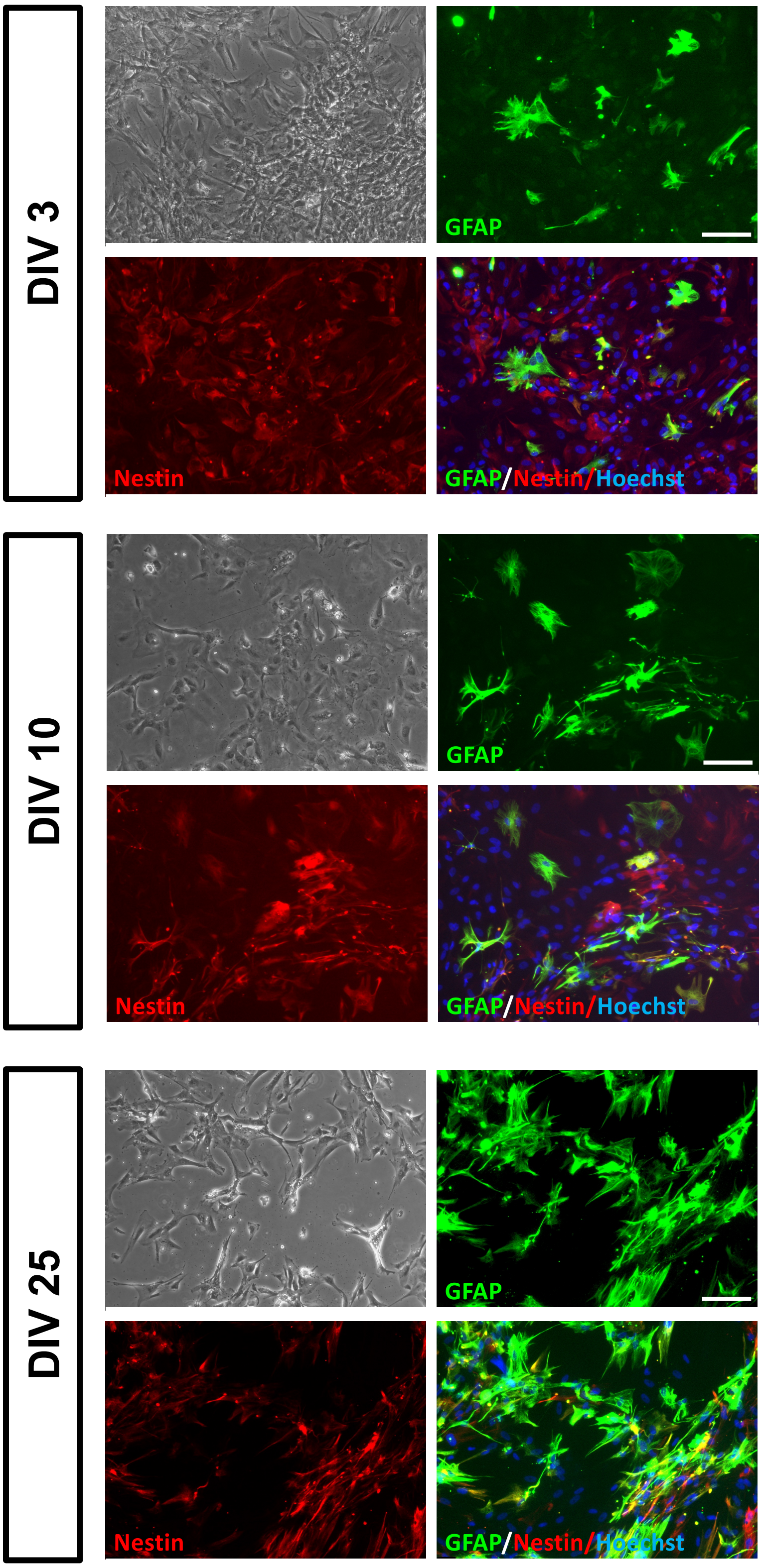


**Supplementary Figure S3.** CX APCs derived in AIM-SFM conditions gradually differentiated into CX Astrocytes upon exposure to AMM-FBS. Representative phase contrast and immunofluorescence pictures of CX APC cultures at DIV 3, 10 and 25 of exposure to AMM-FBS. The fluorescence images are related to the GFAP and Nestin markers and the superimposition of these are shown. Nuclei are stained with Hoechst. 10X magnification. Scale bar: 100 µm.

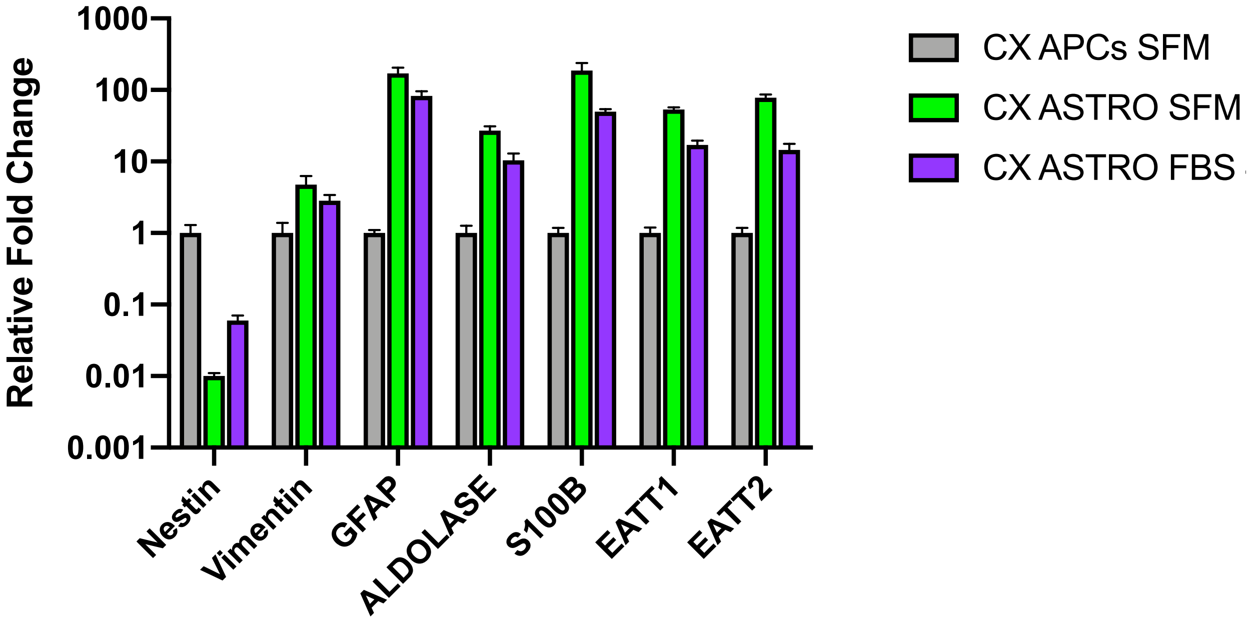


**Supplementary Figure S4.** CX APCs undergo astrocyte differentiation/maturation at DIV 40 of exposure to AMM-SFM and AMM-FBS culture conditions. qPCR analysis for Nestin, Vimentin, GFAP, ALDOLASE, S100B, EAAT1 and EAAT2 transcript levels in hiPSC-derived CX APCs in proliferating SFM conditions and CX APCs at DIV 40 of exposure to astrocyte maturation in AMM-SFM (CX ASTRO SFM) or AMM-FBS (CX ASTRO FBS). Values were normalized to GAPDH and represent the fold change ratio value (log10 scale) related to CX APCs in ACM-SFM (DIV 0). Data are expressed as the means ± STDV (n = 3 biologically independent experiments).
